# Supplementary material for: Parallel encoding of sensory history and behavioral preference during Caenorhabditis elegans olfactory learning
Source: eLife. 2016 Jul 6;5:e14000. doi: 10.7554/eLife.14000 (PMC4935464; doi:10.7554/eLife.14000)
Supplement: Source code 2. — DOI: http://dx.doi.org/10.7554/eLife.14000.019 [file elife-14000-code2.zip › SourceCode_OptogeneticsSuite/image_processing/dxAvi/BaseClasses/Debug/BuildLog.htm]

```
|  |
| --- |
| Build Log |


|  |  |  |
| --- | --- | --- |
|  |  | ``` ------- Build started: Project: BaseClasses, Configuration: Debug|Win32 ------- ``` |


|  |
| --- |
| Command Lines |


|  |  |  |
| --- | --- | --- |
|  |  | ``` Creating temporary file "c:\Dxsdk\Samples\C++\DirectShow\BaseClasses\Debug\RSP000001.rsp" with contents [ /Od /I "." /I "..\..\..\..\include" /D "_DEBUG" /D "WIN32" /D "_LIB" /D "_WIN32_DCOM" /D "DEBUG" /D "WINVER=0x400" /D "_MBCS" /FD /EHsc /RTC1 /MTd /GS /Yu"streams.h" /Fp".\Debug/baseclasses.pch" /Fo".\Debug/" /Fd".\Debug/" /W3 /c /Zi /Gz ".\wxutil.cpp" ".\wxlist.cpp" ".\wxdebug.cpp" ".\winutil.cpp" ".\winctrl.cpp" ".\vtrans.cpp" ".\videoctl.cpp" ".\transip.cpp" ".\transfrm.cpp" ".\sysclock.cpp" ".\strmctl.cpp" ".\source.cpp" ".\seekpt.cpp" ".\schedule.cpp" ".\renbase.cpp" ".\refclock.cpp" ".\pullpin.cpp" ".\pstream.cpp" ".\outputq.cpp" ".\mtype.cpp" ".\dllsetup.cpp" ".\ddmm.cpp" ".\ctlutil.cpp" ".\cprop.cpp" ".\combase.cpp" ".\amvideo.cpp" ".\amfilter.cpp" ".\amextra.cpp" ] Creating command line "cl.exe @"c:\Dxsdk\Samples\C++\DirectShow\BaseClasses\Debug\RSP000001.rsp" /nologo" Creating temporary file "c:\Dxsdk\Samples\C++\DirectShow\BaseClasses\Debug\RSP000002.rsp" with contents [ /Od /I "." /I "..\..\..\..\include" /D "_DEBUG" /D "WIN32" /D "_LIB" /D "_WIN32_DCOM" /D "DEBUG" /D "WINVER=0x400" /D "_MBCS" /FD /EHsc /RTC1 /MTd /GS /Yc"streams.h" /Fp".\Debug/baseclasses.pch" /Fo".\Debug/" /Fd".\Debug/" /W3 /c /Zi /Gz ".\dllentry.cpp" ] Creating command line "cl.exe @"c:\Dxsdk\Samples\C++\DirectShow\BaseClasses\Debug\RSP000002.rsp" /nologo" Creating temporary file "c:\Dxsdk\Samples\C++\DirectShow\BaseClasses\Debug\RSP000003.rsp" with contents [ /OUT:"debug\strmbasd.lib" /NOLOGO ..\..\..\..\lib\strmiids.lib  /nodefaultlib  ".\Debug\amextra.obj" ".\Debug\amfilter.obj" ".\Debug\amvideo.obj" ".\Debug\combase.obj" ".\Debug\cprop.obj" ".\Debug\ctlutil.obj" ".\Debug\ddmm.obj" ".\Debug\dllentry.obj" ".\Debug\dllsetup.obj" ".\Debug\mtype.obj" ".\Debug\outputq.obj" ".\Debug\pstream.obj" ".\Debug\pullpin.obj" ".\Debug\refclock.obj" ".\Debug\renbase.obj" ".\Debug\schedule.obj" ".\Debug\seekpt.obj" ".\Debug\source.obj" ".\Debug\strmctl.obj" ".\Debug\sysclock.obj" ".\Debug\transfrm.obj" ".\Debug\transip.obj" ".\Debug\videoctl.obj" ".\Debug\vtrans.obj" ".\Debug\winctrl.obj" ".\Debug\winutil.obj" ".\Debug\wxdebug.obj" ".\Debug\wxlist.obj" ".\Debug\wxutil.obj" ] Creating command line "lib.exe @"c:\Dxsdk\Samples\C++\DirectShow\BaseClasses\Debug\RSP000003.rsp"" ``` |


|  |
| --- |
| Output Window |


|  |  |  |
| --- | --- | --- |
|  |  | ``` Compiling... dllentry.cpp Compiling... wxutil.cpp wxlist.cpp wxdebug.cpp winutil.cpp winctrl.cpp vtrans.cpp videoctl.cpp transip.cpp transfrm.cpp sysclock.cpp strmctl.cpp source.cpp seekpt.cpp schedule.cpp renbase.cpp refclock.cpp pullpin.cpp pstream.cpp outputq.cpp mtype.cpp Generating Code... Compiling... dllsetup.cpp ddmm.cpp ctlutil.cpp cprop.cpp combase.cpp amvideo.cpp amfilter.cpp amextra.cpp Generating Code... Creating library... ``` |


|  |
| --- |
| Results |


|  |  |  |
| --- | --- | --- |
|  |  | ``` Build log was saved at "file://c:\Dxsdk\Samples\C++\DirectShow\BaseClasses\Debug\BuildLog.htm" BaseClasses - 0 error(s), 0 warning(s) ``` |


|  |
| --- |
|  |
```
