# Supplementary material for: Parallel encoding of sensory history and behavioral preference during Caenorhabditis elegans olfactory learning
Source: eLife. 2016 Jul 6;5:e14000. doi: 10.7554/eLife.14000 (PMC4935464; doi:10.7554/eLife.14000)
Supplement: Source code 2. — DOI: http://dx.doi.org/10.7554/eLife.14000.019 [file elife-14000-code2.zip › SourceCode_OptogeneticsSuite/image_processing/dxAvi/index.html]

A Directshow based AVI reader for Matlab -- works with most codecs and
large AVI files (>2Gb)


### A Directshow based AVI interface for Matlab

This
library contains helper functions to **read**
image frames into Matlab from an AVI

file using DirectShow.

It hence,

a) does
not suffer from the 2Gb file size limit of the VFW based Matlab
aviread

b) handles
most codecs installed on the system

c) has
functions quite similar to aviread

d) but
only works for windows :(

-- Depending on your Matlab version, the .mexw32 files may need to be renamed as
.dll

-- testDxAvi.m is a simple
example, should now pretty much run off the bat.

   The library usage is as follows,

            [avi\_hdl, avi\_inf] = dxAviOpen(avi\_filename);

       pixmap = dxAviReadMex(avi\_hdl, frame\_num);

       img = reshape(pixmap,[avi\_inf.Height,avi\_inf.Width,3]);

       dxAviCloseMex(avi\_hdl);

-- Use mex\_cmd.m if you want to recompile the library

            BaseClasses directory is from
"Direct X 9.0 SDK 2002" (Google should get you this)

            Code here is based on GrabBitmaps.cpp
in DirectShow samples (there are also many many other
awesome samples).

            It is quite weird and disappointing that the new DirectX
SDK do not include these very useful samples.

            This library is only tested with VC++ 7 compiler (run mex -setup for this).

Have Fun!

Ashwin Thangali
-- Feb 2006

 
  
View My Stats
